# Stability, structure and scale: improvements in multi-modal vessel extraction for SEEG trajectory planning

**DOI:** 10.1007/s11548-015-1174-5

**Published:** 2015-04-07

**Authors:** Maria A. Zuluaga, Roman Rodionov, Mark Nowell, Sufyan Achhala, Gergely Zombori, Alex F. Mendelson, M. Jorge Cardoso, Anna Miserocchi, Andrew W. McEvoy, John S. Duncan, Sébastien Ourselin

**Affiliations:** 1 Translational Imaging Group, CMIC, University College London, London, UK; 2 Department of Clinical and Experimental Epilepsy, UCL IoN, London, UK; 3 National Hospital for Neurology and Neurosurgery (NHNN), London, UK

**Keywords:** Computer-assisted planning system, Vessel extraction, Depth electrode insertion, Image-guided surgery, Multi-modal segmentation

## Abstract

**Purpose:**

Brain vessels are among the most critical landmarks that need to be assessed for mitigating surgical risks in stereo-electroencephalography (SEEG) implantation. Intracranial haemorrhage is the most common complication associated with implantation, carrying significantly associated morbidity. SEEG planning is done pre-operatively to identify avascular trajectories for the electrodes. In current practice, neurosurgeons have no assistance in the planning of electrode trajectories. There is great interest in developing computer-assisted planning systems that can optimise the safety profile of electrode trajectories, maximising the distance to critical structures. This paper presents a method that integrates the concepts of scale, neighbourhood structure and feature stability with the aim of improving robustness and accuracy of vessel extraction within a SEEG planning system.

**Methods:**

The developed method accounts for scale and vicinity of a voxel by formulating the problem within a multi-scale tensor voting framework. Feature stability is achieved through a similarity measure that evaluates the multi-modal consistency in vesselness responses. The proposed measurement allows the combination of multiple images modalities into a single image that is used within the planning system to visualise critical vessels.

**Results:**

Twelve paired data sets from two image modalities available within the planning system were used for evaluation. The mean Dice similarity coefficient was $0.89\pm 0.04$, representing a statistically significantly improvement when compared to a semi-automated single human rater, single-modality segmentation protocol used in clinical practice ($0.80 \pm 0.03$).

**Conclusions:**

Multi-modal vessel extraction is superior to semi-automated single-modality segmentation, indicating the possibility of safer SEEG planning, with reduced patient morbidity.

## Introduction

The primary goal of epilepsy surgery is to remove the epileptogenic zone, the minimum amount of cortex that must be resected to produce seizure freedom [3, 16]. As the epileptogenic zone may not be associated with a clear structural abnormality, intracranial electrodes must be used to record the area of the brain where seizures start, known as the seizure-onset zone (SOZ) [3]. Stereo-electroencephalography (SEEG) is the recording of the brain electrical activity by depth electrodes implanted into the brain parenchyma to precisely identify the SOZ. The major complication associated with SEEG implantation is intracranial haemorrhage with a risk that ranges from 0.6 to 2.7 % [13] and reported morbidity and mortality of 5.6 % [2] and 1 % (or less) [13], respectively. To reduce the risk of this and other associated complications, it is necessary to identify electrode trajectories with adequate cortical coverage that pass through safe avascular planes. This is done through pre-operative SEEG planning.

In recent years, there has been great interest in the development of computer-assisted planning systems for optimising intracranial depth electrode insertion [1, 4, 5, 18]. These methods require the effective extraction of critical brain landmarks (such as vessels, ventricles and sulci), with high accuracy and robustness. Despite this requirement, the techniques used to extract the landmarks are very general and may not be the best for this application. While some efforts have been made to improve them [1, 4], greater improvements yet may be obtained using domain-specific knowledge. Furthermore, as pointed by Du et al. [4], the evaluation or validation of the extraction of these brain landmarks is not included in these studies, which hinders the identification of potential problems of the currently used techniques. In this work, we specifically address the extraction of the intracranial vasculature within an SEEG planning system.

A key part of the landmark identification is the vessel extraction. This, despite years of research [9], remains a challenging problem. For this reason, Essert et al. [5], avoid dealing with it directly. Instead, they segment the cortical sulci under the hypothesis that vessels of interest are generally located there. Existing methods of vessel extraction still tend to suffer from discontinuities (caused by low intensity due to partial volume effects and noise) and false-positives; both of these are undesirable as they can lead to invalid or suboptimal trajectories. A common solution, adopted by Bériault et al. [1] and Du et al. [4], is the use of a vesselness filter [6, 10, 17] that enhances voxels within tubular structures. These filters have been very successful thanks to the inclusion of multiple spatial scales within their formulation, but lack information about the surrounding structures. Also, despite increased access to multi-modal images, particularly within computer-assisted planning systems, very few methods have exploited the information complementarity to improve vessel extraction. Passat et al. [15] combined multiple MR sequences to segment the superior sagittal sinus, but their vessel extraction was only performed on a single image, with a second modality used to provide a priori anatomical information of the brain. More recently, Bériault et al. [1] used susceptibility weighted imaging (SWI) and time-of-flight (TOF) images within a planning system to segment veins and arteries, but they do so in a separate fashion and leave it up to the human planner to combine the information from the two modalities. Elsewhere, Hu et al. [8] have proposed a bi-modal approach to 2D retinal vessel extraction using a k-NN classifier trained on features from both modalities.

In this paper, we present a novel method that integrates the concepts of scale, neighbourhood structure and feature stability with the aim of improving the robustness and accuracy of vessel extraction within a computer-assisted SEEG planning system [19]. The method accounts for both the scale and vicinity of a voxel by formulating the problem within a multi-scale tensor voting framework. Feature stability is achieved by introducing a similarity measure that accounts for the multi-modal consistency in the vesselness responses. The proposed measurement enables the combination of multiple responses into a single image that is used within the planning system to visualise critical vessels. This article is an extended version of Zuluaga et al. [20]; it explains the methodology in more detail, extends the original method to cope with an arbitrary number of images and proposes a new way to consider multiple scales within the tensor voting framework to improve the computational speed. A new set of validation experiments is also included.

## Method

The tensor voting framework [11] is a robust technique for extracting structures from a cloud of points. It is based on the principle that a set of unconnected tokens (i.e. points) can exchange information within a neighbourhood that allows one to infer the geometric structure in which a token lies. In 3D, it provides a way to estimate the likelihood that a token lies on a surface, curve or junction (as opposed to being just noise).

**Fig. 1:**
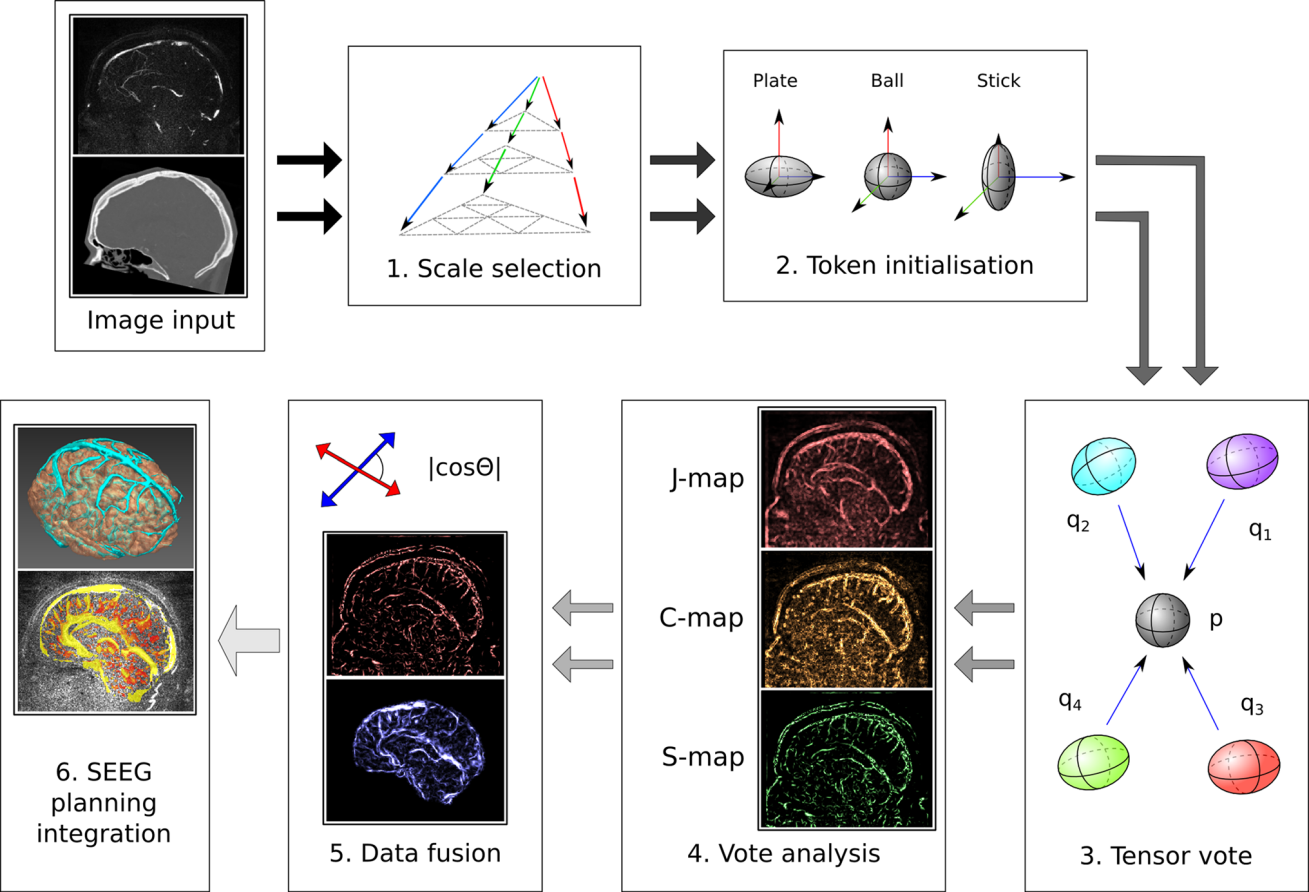
Vessel extraction *diagram*. After optimal scale selection, *images* are converted into tokens through analysis of the Hessian matrix. After voting, the resulting saliency *maps* are combined using the cosine between the vectors defining orientation. The resulting probability *map* is then visualised in the planning system

Tensor voting consists of three stages: token initialisation, where token locations are identified and their tensors assigned; tensor voting, where tensors from a token and its neighbours are combined; and analysis of voting results. In order to give our method feature stability and scale invariance, we add a data fusion step and then embed the different components into a multi-scale framework. A diagram illustrating the proposed method is shown in Fig. 1.

### Multi-scale framework and voxel selection

In previous work [20], we formulated the tensor voting approach into a multi-scale framework by evaluating the response of different scales at the data fusion stage and retaining the maximum response through scales. This consists in performing steps 2–5 in Fig. 1 for every scale and image modality. As the tensor vote has to be computed every time, this can be very computationally intensive. To reduce the computational burden of the method, we now perform the multi-scale analysis before the tensor voting step, rather than at the final stage.

Let $I(\mathbf {x})$ be an image and $H_{\sigma }(\mathbf {x})$ be the Hessian matrix at voxel $\mathbf {x}$ at scale $\sigma$. By means of the eigenvalues $\kappa _{i}$ and eigenvectors $\mathbf {v_{i}}$ of $H_{\sigma }(\mathbf {x})$, a vesselness function $\nu (\mathbf {x},\sigma )$ [10] is computed at a range of scales $[\sigma _\mathrm{min} ,\sigma _\mathrm{max}]$. An “optimal scale” image $M(\mathbf {x})$ is constructed by finding the value of $\sigma$ with the maximum vesselness response at each voxel:

1 $$\begin{aligned} M(\mathbf {x})=\sigma ^{*},&\quad \sigma ^{*}=\mathop {{{\mathrm{arg\,max}}}}\limits _{\sigma _\mathrm{min}\le \sigma \le \sigma _{max}} \nu (\mathbf {x},\sigma ). \end{aligned}$$

While the Hessian and its eigensystem are computed across the full range of scales, all further calculations involving scale analysis are performed using the scale defined by $M(\mathbf {x})$. For simplicity, we denote the Hessian at the locally optimal scale as $H(\mathbf {x})$.

We derive from this eigensystem the information about which voxels are likely to belong to a vessel structure. The eigenvalues of $H(\mathbf {x})$ are ordered by their absolute value, so that $|\kappa _{3}| \ge |\kappa _{2}| \ge |\kappa _{1}|$. For bright vessels on a dark background, a voxel is expected to belong to a vessel only if $\kappa _{2} < 0$ and $\kappa _{3} < 0$ [10]. Based on this information, voxels that do not satisfy this condition for at least one scale can be discounted in the further analysis. Voxels satisfying the condition go on to take part in the tensor voting, with their Hessian computed at the locally optimal scale. Under the voting formalism, a voxel $\mathbf {x}$ considered for tensor voting is called a token and is denoted as $\mathbf {p}$.

### Token initialisation

The information contained in a token $\mathbf {p}$ is encoded on a 3D second-order, symmetric, nonnegative definite tensor $\mathbf {T}$. According to the spectrum theorem, $\mathbf {T}$ can be expressed as the linear combination of three tensors:

2 $$\begin{aligned} \mathbf {T} \!= (\lambda _{1}-\lambda _{2})(\mathbf {e_{1}}\mathbf {e_{1}}^{T}) +(\lambda _{2}-\lambda _{3}) \sum _{i=1}^{2}\mathbf {e_{i}}\mathbf {e_{i}}^{T} \!+\lambda _{3}\sum _{i=1}^{3}\mathbf {e_{i}}\mathbf {e_{i}}^{T}, \end{aligned}$$

where $\lambda _{i}$ are the eigenvalues obtained from the eigendecomposition of $\mathbf {T}$, with $\lambda _{1}> \lambda _{2}> \lambda _{3}\ge 0$, and $\mathbf {e_{i}}$ are the corresponding eigenvectors.

While the tensor is most commonly expressed as a $3\times 3$ matrix, it can be viewed as a 3D ellipsoid whose shape describes the contribution of its different components in Eq. 2. These components are known as the stick tensor $S$, the plate tensor $P$ and the ball tensor $B$. More precisely, through expansion of the second and third terms, they are defined as:

3 $$\begin{aligned} S&=\left( \lambda _{1}-\lambda _{2}\right) \left( \mathbf {e_{1}}\mathbf {e_{1}}^{T}\right) \nonumber \\ P&=\left( \lambda _{2}-\lambda _{3}\right) \left( \mathbf {e_{1}}\mathbf {e_{1}}^{T}+\mathbf {e_{2}}\mathbf {e_{2}}^{T}\right) \nonumber \\ B&=\lambda _{3}\left( \mathbf {e_{1}}\mathbf {e_{1}}^{T}+\mathbf {e_{2}}\mathbf {e_{2}}^{T}+\mathbf {e_{3}}\mathbf {e_{3}}^{T}\right) . \end{aligned}$$

Each tensor component corresponds to a different type of structural information: the stick tensor represents an elongated ellipsoid encoding eccentricity with orientation $\mathbf {e_{1}}$, the plate tensor represents a disc-shaped structure with normal $\mathbf {e_{3}}$, and the ball tensor represents a round structure in which all orientations are equally probable. The scalar values associated with each tensor are the saliency measurements of “surfaceness” $(\lambda _{1}-\lambda _{2})$, “curveness” $(\lambda _{2}-\lambda _{3})$ and “junctionness” $\lambda _{3}$. Points with very small eigenvalues are regarded as noise.

The scalar information contained in a greyscale image needs to be encoded into a tensor that satisfies Eqs. 2–3 before it can be used within the tensor voting framework. Depending on which tensor is assigned to the tokens in the token intitialisation step, different aspects of image will inform the final vessel segmentation. In this paper, we explore the three alternatives presented below.

#### No preferred orientation

A common approach is to assign an isotropic, ball-shaped tensor to each of the tokens [11, 12]. All three eigenvalues of a token’s tensor have the same value, $K$. While the tensors at each token have the same shape, they can be given different magnitudes (values of $K$) according to their tokens’ vessel salience score. In this way, tokens that are most likely to be on the vessels are given greater importance in the final segmentation. In this work, we experiment with using both a token’s vesselness, $\nu (\mathbf {p})$, or its image intensity, $I(\mathbf {p})$, as salience score/weight.

#### Hessian matrix-based orientation

The analysis of the eigensystem of the Hessian matrix provides information about the orientations of structures within an image [6]. Let us recall $\mathbf {v_{i}}$, the eigenvectors of $H(\mathbf {p})$ at the optimal scale, and $|\kappa _{3}| \ge |\kappa _{2}| \ge |\kappa _{1}|$, its eigenvectors. Tokens are initialised with a stick tensor $S$ that has orientation $\mathbf {v_{1}}$ (the eigenvector associated with $\kappa _{1}$), the direction along the vessel. The saliency of the stick tensor is obtained by assigning the $\kappa _{i}^{-1}$ to the corresponding $\lambda _{i}$. As with the ball tensor, it is possible to initialise the stick tensors with different weights reflecting an initial estimate of a token’s vesselness. Section “Experiments and results” compares the results obtained when varying these values.

*Structure tensor-based initialisation.* Given $\bigtriangledown I(\mathbf {p})$,^1^ the image gradient at a given token position $\mathbf {p}$, Moreno et al. [12] showed that the tensorised gradient, $\bigtriangledown I \bigtriangledown I^{T}$, can be used to initialise a stick tensor $S$ at every token, giving a saliency measurement $\Vert \bigtriangledown I \Vert ^{2}$.

Table 1 summarises the values that are assigned to the tensor’s eigenvalues, $\lambda _{i}$, and eigenvectors, $\mathbf {e_{i}}$, depending on the type of used initialisation.

**Table 1:** Eigenvalues $\lambda _{i}$ and eigenvectors $\mathbf {e_{i}}$ of $\mathbf {T}$ for each initial orientation initialisation

Initialisation	Eigenvalues $\lambda _i$	Eigenvectors $\mathbf {e_{i}}$
No preferred orientation	1: $\lambda _{1}=\lambda _{2}=\lambda _{3}=K$	$\mathbf {e_{i}}=(e_{ij})$, $e_{ij}=\delta _{ij}$
	2: $\lambda _{1}=\lambda _{2}=\lambda _{3}=I(\mathbf {p})$	
	3: $\lambda _{1}=\lambda _{2}=\lambda _{3}=\nu (\mathbf {p})$	
Hessian matrix	1: $[\lambda _{1}, \, \lambda _{2}, \, \lambda _{3}] =[|\kappa _{1}|^{-1},|\kappa _{2}|^{-1},|\kappa _{3}|^{-1}]$	$[\mathbf {e_{1}}, \,\mathbf {e_{2}}, \,\mathbf {e_{3}}] = [\mathbf {v_{1}}, \,\mathbf {v_{2}}, \,\mathbf {v_{3}}]$
	2: $[\lambda _{1}, \, \lambda _{2}, \, \lambda _{3}] =[\nu (\mathbf {p}),0,0]$	
Structure tensor	$(\lambda _{1}-\lambda _{2})=\Vert \bigtriangledown I \Vert ^{2}$, $\lambda _{3}=0$	$\mathbf {e_{1}}\mathbf {e_{1}}^{T}= \bigtriangledown I \bigtriangledown I^{T}$, $\mathbf {e_{2}}=\mathbf {e_{3}}=\mathbf {0}$

Note that when no preferred orientation is used, three different saliency measurements can be used. Similarly, two different configurations of the initial eigenvalues are proposed when using Hessian matrix analysis

### Tensor voting

After each token $\mathbf {p}$ is encoded as a tensor $\mathbf {T}$ in the form of Eq. 2, it propagates structural information to its neighbours in the form of a vote. Votes are combined through addition at every token to infer the type of structure going through it. More formally, the tensor voting at $\mathbf {p}$ is given by [12]:

4 $$\begin{aligned} TV\left( \mathbf {p}\right) = \sum _{\mathbf {q}\in \chi }\left( \mathrm{SV}\left( \mathbf {v},S_{\mathbf {q}}\right) +PV\left( \mathbf {v},P_{\mathbf {q}}\right) +BV\left( \mathbf {v},B_{\mathbf {q}}\right) \right) ,\nonumber \\ \end{aligned}$$

where $\chi$ denotes the neighbourhood of $\mathbf {p}$, $\mathbf {q}$ a point belonging to $\chi$, SV, PV and BV the stick, plate and ball votes cast to $\mathbf {p}$ by each component $S_{\mathbf {q}}$, $P_{\mathbf {q}}$, $B_{\mathbf {q}}$ of $\mathbf {q}$ and $\mathbf {v}=\mathbf {p}-\mathbf {q}$. The strength of the vote will be dependent on the norm of $\mathbf {v}$, as the influence of a point $\mathbf {q}$ should decay as its distance from $\mathbf {p}$ increases. Here, $q\in \chi$ is defined as the window of size $N_{\mathbf {p}}$ at every token with

5 $$\begin{aligned} N_{\mathbf {p}}=2M(\mathbf {p}), \end{aligned}$$

where $M(\mathbf {p})$ is the “optimal scale” image defined in Eq. 1.

The derivation of SV, PV and BV in Eq. 4 is extensive and beyond the scope of this paper. We therefore only present their formulation (see Appendix 6), mentioning that the tensor voting procedure can be regarded as a tensor convolution with a voting kernel which itself produces a tensor. The interested reader is referred to [11, 12] for further details.

### Voting analysis

As the result of the tensor voting is another tensor, it can be decomposed as in Eq. 2. From this decomposition, three feature vector maps, the surface (S-Map), the curve map (C-Map) and the junction map (J-Map), are constructed. A voxel of these maps contains a 2-tuple $(s,\mathbf {w})$, where $s$ is a scalar indicating strength/saliency and $\mathbf {w}$ is a unit vector indicating direction. Table 2 summarises the values for $(s,\mathbf {w})$ within the different feature maps.

In the context of our problem, we are interested in the information provided by the S-map (first term of Eq. 2). For a given tuple, we interpret $s$ as a consensus measurement of vesselness between a voxel and its neighbours and $\mathbf {w}$ as the direction along the vessel.

**Table 2:** Feature maps 2-tuple $(s,\mathbf {w})$ definition

Map	$s$	$\mathbf {w}$
S-Map	$\lambda _{1}-\lambda _{2}$	$\mathbf {e_{1}}$
C-Map	$\lambda _{2}-\lambda _{3}$	$\mathbf {e_{3}}$
J-Map	$\lambda _{3}$	Arbitrary

### Data fusion

Given two $D$-dimensional vectors, the cosine of the angle between them is an index on the extent to which they are aligned. As vessels are well-oriented structures, the cosine of the direction vectors is a surrogate of vesselness consistency between different images. Given two sets of tuples $(s_{1},\mathbf {w_{1}})$ and $(s_{2},\mathbf {w_{2}})$ from vesselness maps obtained from two different modalities after voting analysis, with $\Vert \mathbf {w_{1}}\Vert =\Vert \mathbf {w_{2}}\Vert =1$, it is possible to fuse the maps into a single one through the following expression:

6 $$\begin{aligned} \varphi (\mathbf {p})=0.5|\mathbf {w_{1}}\cdot \mathbf {w_{2}}|(s_{1}+s_{2}), \end{aligned}$$

where $\cdot$ denotes the dot product between the two vectors. By refactoring Eq. 6, the fusion can be extended to $L$ different image modalities through:

7 $$\begin{aligned} \varphi (\mathbf {p})=\dfrac{1}{L}\sum \limits _{i=1}^{L}s_{i}\sum \limits _{j=1}^{L} (1-\delta _{ij}) |\mathbf {w_{i}}\cdot \mathbf {w_{j}}|. \end{aligned}$$

The fusion scheme is a measure that rewards consensus and punishes discord; the greater the angular distance between the different directions, the more the absolute value of the resulting output is reduced. When there is complete agreement between modalities, the output is simply the average. When their directions are perpendicular, the output drops to zero to reflect a complete lack of certainty.

### SEEG planning system integration

Similar to Bériault et al. [1] and Du et al. [4], the resulting vessel probability map, $\varphi (\mathbf {p})$, is used as input of our computer-assisted planning system [19]. As the electrode-implanting trajectory needs to be further than a safety margin from the critical tissue (vessels in this case), the probability map serves as a measure of risk of crossing a vessel. Within the planning system, the probability map is converted into a 3D surface mesh object, coloured using a pre-defined landmark colour scheme [19] and displayed within the neuronavigation planning system along with other brain structures (Fig. 3).

## Experiments and results

### Data

Blood vessel images for the computer-assisted planning system [19] were acquired using 3D phase contrast MRI (3DPC) and CT angiograms (CTAs). For our experiments, we used twelve paired data sets of 3DPC and CTA available within the planning system (informed consent obtained from all the patients). The 3DPC data were acquired on a 1.5-T Siemens Avanto MR scanner with voxel size resolution $0.8593\times 0.8593\times 1\,\hbox {mm}^{3}$ and velocity encoding of 5 cm/s in each direction. CTA images were acquired on a Siemens SOMATOM Definition AS+ scanner with voxel size resolution $0.4296\times 0.4296\times 0.75\,\hbox {mm}^{3}$. During image acquisition, the patient’s head was immobilised by placing a pad between the head and the coil.

### Gold standard generation

Three different observers (a neurosurgical trainee, a physicist with 8-year experience in clinical neuroimaging and a master student trained for the task) segmented blood vessels structures following the protocol typically used in clinical practice for SEEG planning in the absence of a computer-assisted planning system. The semi-automated annotation procedure followed the steps described hereafter:

1. An intracranial space mask was applied to the CTA image to remove skull and to the 3DPC image to remove extracranial blood vessels.
2. Masked CTA and 3DPC were separately thresholded to give an initial estimate of the vessels. The threshold was defined by visually evaluating the resulting segmentation and determining whether noise and blood vessels were easy to distinguish and differentiate from each other with minimal manual cleaning.
3. Small isolated clusters were removed based on diameter size within MeshLab. The observers varied the threshold until they considered the segmented result satisfactory through visual inspection. Afterwards, large noise (e.g. calcifications) was removed by manually editing the images using MeshLab.

The six segmentations of the observers were combined into a consensus agreement through a voting strategy in a similar fashion as Hameeteman et al. [7].

### Validation scheme

The proposed algorithm was evaluated on the twelve affinely co-registered [14] data sets using ten different scales between $\sigma _\mathrm{min} = 1.0$ and $\sigma _\mathrm{max} =4.5$ distributed equally in a log space.

For a quantitative evaluation, each of the twelve vessel images $S$ was compared to the consensus agreement $M$ using the Dice similarity coefficient (DSC):

8 $$\begin{aligned} \mathrm{DSC}=\dfrac{2 \times |S \cap M|}{|S+M |}, \end{aligned}$$

where the intersection operation is the voxel-wise minimum operation and $|\cdot |$ is the integration of the voxel values over the complete image [7].

We used the DSC to assess the performance of our method and that one of each observer w.r.t the consensus when doing a semi-automated segmentation with a single modality as is done in clinical routine. We could then compare the accuracy of the proposed method to current practice.

### Evaluation of different initialisation strategies

In Section “Token initialisation”, we presented different strategies to initialise the token’s tensors. Initialisation involves the definition of an initial orientation and the saliency of the tensors. In order to determine the effects of initialisation in vessel extraction, we evaluated the performance of the six different initialisation configurations (Table 1).

#### No preferred orientation

Three different saliency measurements were estimated to initialise ball tensors: a constant value for every tensor $K=1$, the image intensity at each token, $\lambda _{1-3}=I(\mathbf {p})$, and a vesselness measurement at each token $\lambda _{1-3}=\nu (\mathbf {p})$. We chose the vesselness measurement proposed by Manniesing et al. [10] due to its smoothness properties. In order to compute the vesselness function, we followed the guidelines reported in the original publication [10].

Reported DSCs were $0.42 \pm 0.04$, $0.79 \pm 0.03$ and $0.89 \pm 0.04$ for constant value, intensity-based and vesselness measurement initialisation, respectively. Not surprisingly, the use of a constant value to initialise the saliency gives the worst results. This shows that the use of a priori information to define initial saliency improves the vessel extraction quality.

#### Hessian-based analysis initialisation

Two different initialisation schemes based on the analysis of the Hessian matrix were evaluated: the use of the eigenvalues $\kappa _{i}$ of $H$, and a modified scheme to reflect a token’s vesselness. For this second approach, we assigned the response of vesselness filter [10], i.e. $\lambda _{1}=\nu (\mathbf {p})$, while the other two $\lambda$ values were set to zero. The DSC obtained using the response of vesselness filter ($0.88 \pm 0.04$) was higher than the one obtained by directly using the $\kappa _{i}$ of the Hessian matrix ($0.76 \pm 0.05$).

#### Structure tensor-based initialisation

Tokens initialised through the tensorised image gradient were assigned a saliency measurement equal to the squared gradient magnitude $\Vert {\bigtriangledown }I \Vert ^{2}$. The value used for the Gaussian kernel size involved in the computation of the structure tensor was obtained from the multi-scale analysis. A DSC of $0.65 \pm 0.04$ was reported.

#### Summary

**Fig. 2:**
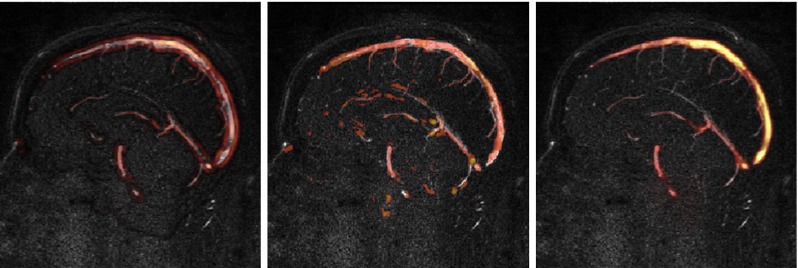
Probability maps $\varphi$ obtained using, from *left* to *right*, no preferred orientation, Hessian-based analysis and structure tensor-based initialisation. The response of a vesselness filter [10] was used as initial saliency measurement for the first two cases. The approaches using the response of the vesselness filter are more sensible to fine structures. The structure tensor approach fails to detect small vessels, but has a strong response in large vessels

Table 3 summarises the mean Dice score coefficients obtained when comparing the proposed method, using the three different initialisation strategies (no preferred orientation, Hessian-based and structure tensor-based), to the consensus $M$. For no preferred orientation and Hessian-based initialisation schemes, only the best performing configuration is reported (i.e. using the vesselness filter result). The obtained DSCs using no orientation ($0.89 \pm 0.04$) and Hessian-based initialisation ($0.88 \pm 0.04$) indicate similar performance. Performance using structure tensor initialisation, on the other hand, appears to be worse ($0.65 \pm 0.04$). A visual inspection of the probability maps $\varphi$ obtained through each strategy (Fig. 2) showed that structure tensor-based initialisation tends to extract big vessels, but fails in the extraction of the small ones, explaining its lower DSC.

**Table 3:** Dice score coefficients obtained using the three different initialisation strategies: no preferred orientation, Hessian-based analysis and structure tensor-based initialisation

	No orientation	Hessian analysis	Structure tensor
DSC	$0.89\pm 0.04$	$0.88\pm 0.04$	$0.65\pm 0.04$

### Multi-modal versus single-modality extraction

To evaluate the relevance of the multi-modal approach, we compared the proposed method with the results obtained when using a single modality. We evaluate the results obtained when combining the two modalities through:

9 $$\begin{aligned} \varphi _\mathrm{min}(\mathbf {p})= & {} \min (s_{1},s_{2}) \nonumber \\ \varphi _\mathrm{max}(\mathbf {p})= & {} \max (s_{1},s_{2}). \end{aligned}$$

It should be noted that the $\min$ and $\max$ operations are used instead of the logical AND and OR operators, since the images are probabilistic, rather than binary maps.

Results show that when using a single image modality, CTA tends to perform better than 3DPC (Fig. 4). By combining the modalities through the $\min$ and $\max$ operations, the performance w.r.t 3DPC is improved, but it degrades performance of CTA. To understand this behaviour, we visually inspected the maps obtained through both methods. When using $\min$, weak vesselness responses and discontinuities are propagated into the final map. On the other hand, $\max$ favours continuity, but at the cost of increasing the number of false-positives mainly close to boundaries. As a result of this, the DSC can drop dramatically in some cases (see outliers for max in Fig. 4). The proposed method offers a productive balance between the two: False-positives diminish where there is no directionality agreement and weak vesselness responses are not suppressed.

**Fig. 3:**
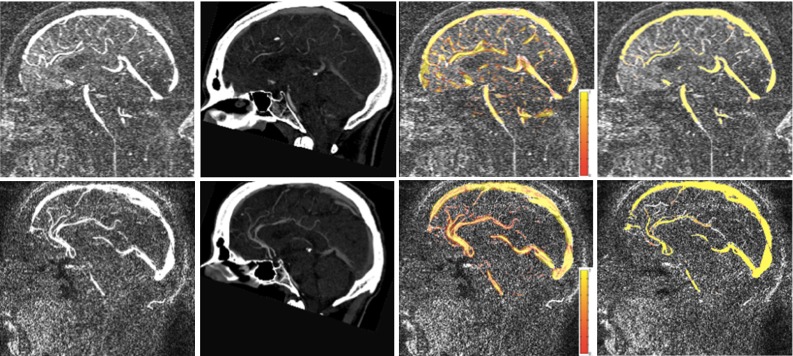
3DPC (*first column*) and CTA images (*second column*), superposed vesselness map generated by the proposed method over 3DPC (*third column*) and consensus for two subjects (*fourth column*)

### Multi-modal extraction versus single-modality observers

**Fig. 4:**
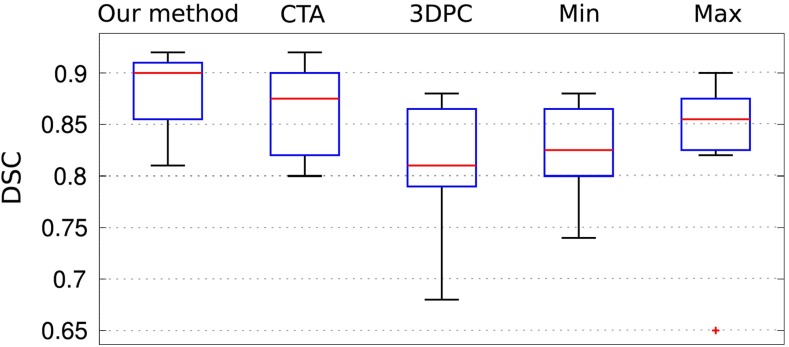
*Boxplots* displaying the DSC for the proposed method, the single-modality results (without data fusion) using CTA and 3DPC, and data fusion through $\min$ and $\max$ operators. The *red cross* represents an outlier

The mean Dice coefficients obtained when comparing our method and the observer’s annotations to the consensus $M$ are summarised in Table 4. The DSC of the proposed multi-modal approach is superior to the one obtained by the best performing rater using a single modality. Although CTA images have richer vessel content that is reflected in better rater segmentations, 3DPC contains complementary information that is exploited by the proposed algorithm. A visual comparison of obtained vesselness maps with a consensus map is given in Fig. 3 to further illustrate the performance of our method w.r.t. the current semi-automated approach (Fig. 4).

**Table 4:** Mean $\pm$ standard deviation of the Dice similarity coefficient (DSC) when comparing our method and the observers annotations to the consensus agreement

	Our method	Observer 1		Observer 2		Observer 3	
		3DPC	CTA	3DPC	CTA	3DPC	CTA
DSC	$0.89\pm 0.04$	$0.35\pm 0.10$	$0.76\pm 0.04$	$0.37\pm 0.10$	$0.80\pm 0.03$	$0.37\pm 0.09$	$0.79\pm 0.03$

### Computational time

**Fig. 5:**
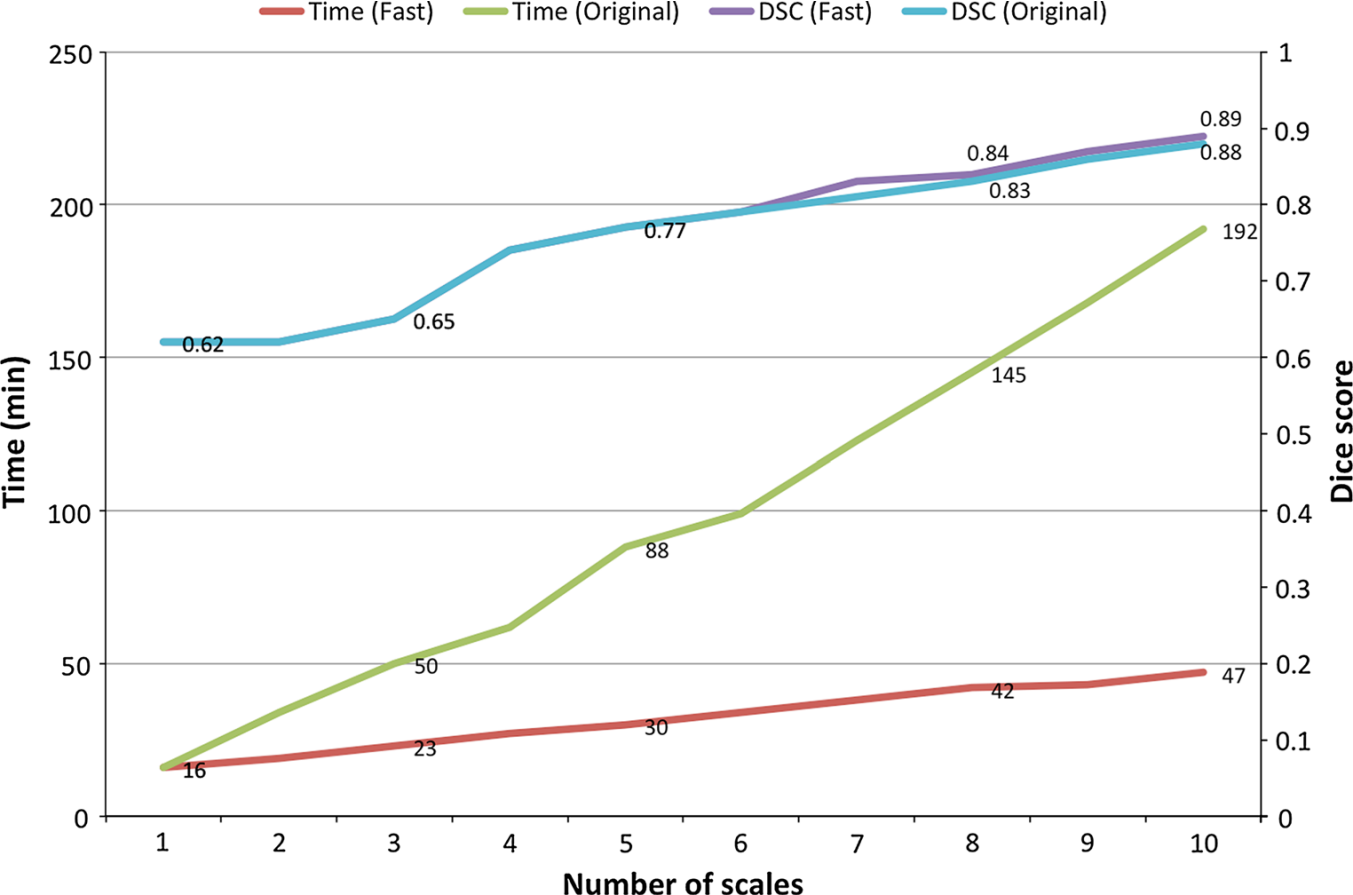
Average execution times of the proposed approach (fast) and our original formulation Zuluaga et al. [20] as a function of the number of scales. Dice score coefficients (DSCs) for both methods are also displayed to show that speedup of the method is not at the cost of accuracy

To demonstrate the improvement in computational time due to the modification of the multi-scale analysis, we compared the execution times of the current method and the original one [20] as a function of the number of scales. Both methods were executed on a PC with a quad-core processor (2.13 GHz). Figure 5 shows the reported execution times along with the Dice score coefficients obtained by each method. Results show that the computational time of the new approach decreases at no cost to the method’s accuracy, which remains unchanged.

### Integration into the SEEG planning system: visual assessment

**Fig. 6:**
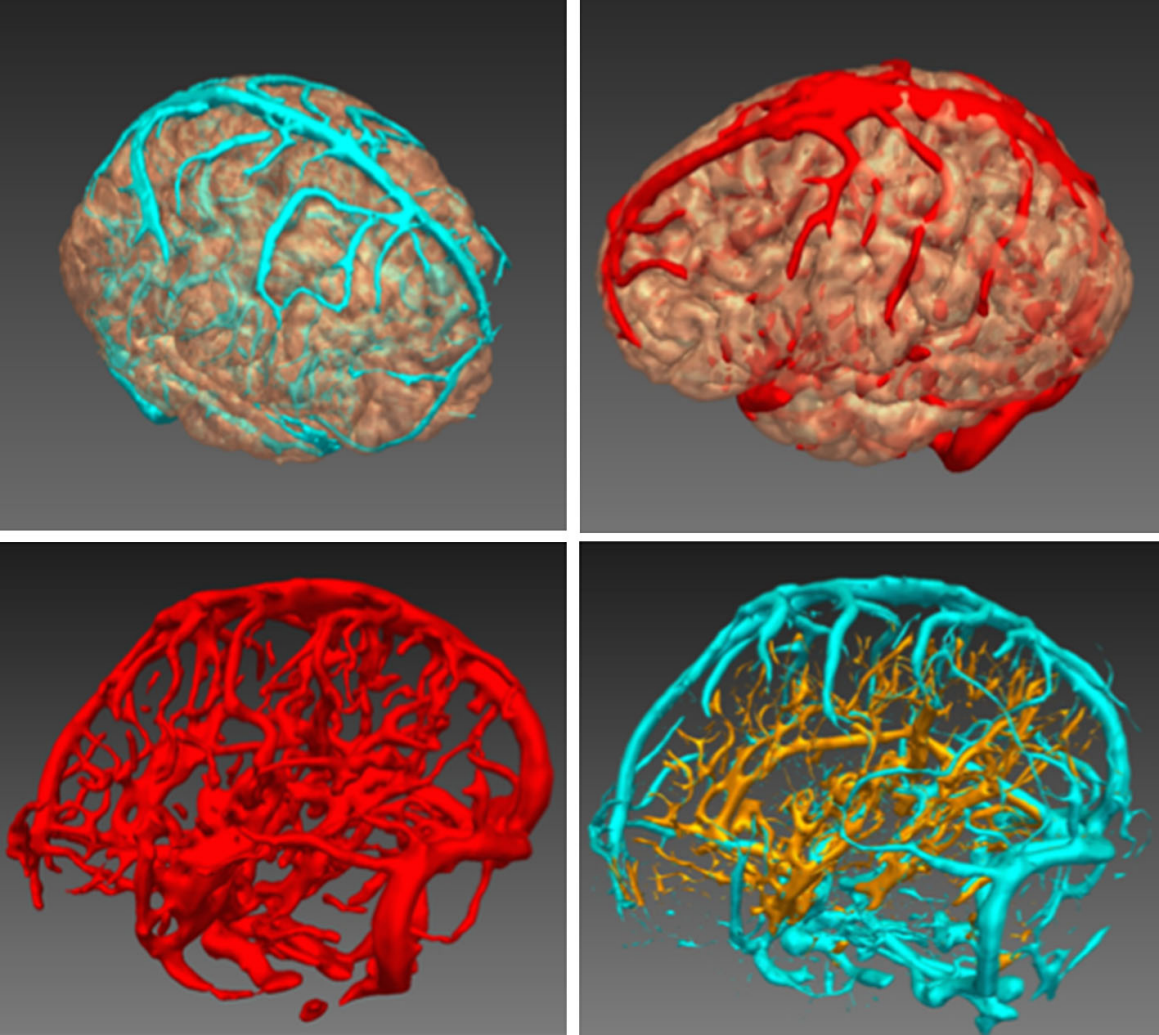
Integration into the computer-assisted planning system. On *top*, examples of displayed extracted vessels using different *colour schemes*. On *bottom left*, display of a segmented vascular tree contrasted with the combined single-modality segmentation, *right*, from 3DPC (*blue*) and CTA (*gold*). The results obtained with the proposed method contain less noise

Figure 6 presents different examples of the visualisation within the SEEG planning system. Furthermore, we compare the results obtained with the proposed method (Fig. 6 bottom, left) with the extracted vessels when 3DPC and CTA are processed separately (Fig. 6, bottom, right). The images show how the developed method combines the information coming from both modalities while remaining robust to noise.

## Discussion

Brain vessels are among the most critical landmarks that need to be assessed in order to mitigate surgical risks [4]. Traditionally, the procedure of extracting vessels (and other structures) is performed in a semi-automated manner by an expert inspecting an image. This is time-consuming, difficult and prone to errors due to the complexity of the vessel network. The development of fully automated and robust methods that can reduce the workload is highly desirable. Here, we have presented a fully automated method that integrates scale, neighbouring structure and feature stability within a single framework to improve vessel extraction within an SEEG planning system.

The proposed method is built upon the tensor voting framework [11]. We have extended it by introducing the evaluation of multiple scales and by using complementary sources of information to reduce noisy structures and to improve the connectivity of voxels. Although we have evaluated the proposed framework with two image modalities in this work, its formulation is generic enough that it can be applied to any number of modalities.

The tensor voting framework requires encoding of greyscale information into a tensorised form. We have evaluated a set of different alternatives for tensor initialisation, which include the use of no priors, zero-order (intensity), first-order (the structure tensor) and second-order information (Hessian-based). Our results have shown that the information derived from the analysis of the Hessian matrix, when used to initialise saliency, provides the best results. Results also show that the selection of optimal saliency measures is more critical than initial tensor orientations. The Dice score coefficients obtained when using vesselness measures as saliency were the highest, independently of the tensor orientation used (no orientation or Hessian-based). On the other hand, changes in the type of saliency measurement used for initialisation did greatly influence the extraction accuracy.

By comparing our method with its single-modality equivalent (e.g. tensor voting without data fusion), we have shown that the use of multiple image types increases performance. This is simply explained by the complementary information offered by the different images. We have also found that the way the information from all sources is combined influences the algorithmic performance. Through the use of a priori knowledge on tubular structures, we favour the fusion of well-aligned objects. Many of the previously seen false-positives are absent form the final probability map as a result, particularly along the brain boundaries.

It is common to find weak vesselness responses in different regions of the vessel tree in both 3DPC and CTA, and discontinuities are likely to appear there if a single modality is used for visualisation. By combining several modalities, the response of weak vessels increases, improving the continuity of the vessel tree and its visualisation within the planning system. The improvement in the continuity of the vessel tree, combined with a decrease in false-positives detected, should lead to a better path planning.

The comparison of the proposed method with human raters has shown that the use of combined image modalities represents an advantage w.r.t current practice. The presented results are more accurate than a human observer using a single modality. However, the method has some limitations that need to be solved before it can be deployed in clinical practice. While discontinuities are reduced w.r.t single-modality segmentation, they can still appear in small branches. Following the principle of using complementary sources of information (i.e. different image modalities of the same object/anatomical structure), a natural extension of the presented approach to solve this limitation is to combine the outputs obtained when processing different sources of information (e.g. zero-, first- and second-order information or S-,C- and J-maps) into a single probability map. As an example, the structure tensor provides the best results on larger vessels but not performs poorly on small vessels (Fig. 2). Under this scenario, the best features of each source of information would be exploited instead of just keeping the one that overall performs best.

Regarding computational time, which is a key feature if an algorithm is to be translated into the clinic, we have reformulated the multi-scale analysis to reduce the time required to extract the vessel tree. The results show that the new formulation is nearly four times faster than the original one [20] at no cost for the vessel extraction accuracy.

## Conclusions

In this paper, we have presented a vessel extraction method for the identification of critical landmarks within a computer-assisted SEEG planning system. The main feature of this method is that it integrates scale, neighbouring structure and feature stability within a single framework. The introduction of a voting neighbourhood within the well-established multi-scale approach and the use of complimentary sources of information reduces noisy structures and improves the connectivity of the voxels belonging to vessels. The results presented here demonstrate the superiority of our method to the semi-automated single-modality segmentation, indicating the possibility of safer SEEG planning with reduced patient morbidity.

## Appendix: Stick, plate and ball tensor voting

For the sake of completeness, we present in this section the expressions for SV, PV and BV, the stick, plate and ball votes. Further details on the mathematical derivation of the three expressions can be found in [11, 12].

### Stick tensor vote

Given $S_{\mathbf {q}}$, which encodes the orientation of the normal at point $\mathbf {q}$, a vote casted from $\mathbf {q}$ to $\mathbf {p}$ can be defined as:

10 $$\begin{aligned} \mathrm{SV}(\mathbf {v},S_{\mathbf {q}})=s_{1s}\left[ R_{2\theta } \,\,\, S_{\mathbf {q}} \,\,\, R^{T}_{2\theta }\right] , \end{aligned}$$

where $\theta$ represents the angle defining the orientation of $\mathbf {v}$, $R_{2\theta }$ is a rotation w.r.t the axis $\mathbf {v} \times (S_{\mathbf {q}}\mathbf {v})$, and $s_{1s}$ is a decaying function used to weight the vote and defined as:

11 $$\begin{aligned} s_{1s}(\mathbf {v},S_{\mathbf {q}})=\left\{ \begin{array}{ll} e^{-\frac{l^{2}+b\kappa ^{2}}{\rho ^{2}}} &{} : \theta \le \pi /4\\ 0 &{} : \text {otherwise}, \end{array}\right. \end{aligned}$$

where $l$ denotes the arc length, $\kappa$ the curvature, $\rho$ is a scale parameter associated with the window size $N_{\mathbf {p}}$, and $b$ is a parameter that can be adjusted to give more importance to the curvature ($b=\Vert \mathbf {v}\Vert ^{2}/4$, [12]).

### Plate tensor vote

Given $S_{P_{\mathbf {q}}}(\beta )$, a stick inside a plate $P$, a plate vote is defined as the aggregation of stick votes cast by all the stick tensors $S_{P_{\mathbf {q}}}(\beta )$ that constitute $P_{\mathbf {q}}$. It is defined as:

12 $$\begin{aligned} \mathrm{PV}(\mathbf {v},P_{\mathbf {q}})=\dfrac{\lambda _{1P_{\mathbf {q}}}}{\pi }\int _{0}^{2\pi } \mathrm{SV}(\mathbf {v},S_{P_{\mathbf {q}}}(\beta ))\mathrm{d}\beta . \end{aligned}$$

### Ball tensor vote

Let $S_{B}(\phi ,\psi )$ be a unitary stick tensor expressed in spherical coordinates with orientation $(1,\phi ,\psi )$. Similar to the plate tensor vote, a ball vote can be obtained through the aggregation of the stick votes within the surface $\Gamma$ of the unitary sphere:

13 $$\begin{aligned} \mathrm{BV}(\mathbf {v},B_{\mathbf {q}})=\dfrac{3\lambda _{1B_{\mathbf {q}}}}{4\pi }\int _{\Gamma }\mathrm{SV}(\mathbf {v},S_{B_{\mathbf {q}}}(\phi ,\psi ))\mathrm{d}\Gamma . \end{aligned}$$

## Footnotes

1 For the sake of simplicity, we will drop the index term and the image gradient at a given token position $\mathbf {p}$ will be expressed as $\bigtriangledown I$ for the remaining of this article.
